# Acknowledgement to Reviewers of *Polymers* in 2017

**DOI:** 10.3390/polym10010069

**Published:** 2018-01-13

**Authors:** Polymers Editorial Office

**Affiliations:** MDPI AG, St. Alban-Anlage 66, 4052 Basel, Switzerland

Peer review is an essential part in the publication process, ensuring that *Polymers* maintains high quality standards for its published papers. In 2017, a total of 755 papers were published in the journal. Thanks to the cooperation of our reviewers, the median time to first decision was 15 days and the median time to publication was 37 days. The editors would like to express their sincere gratitude to the following reviewers for their time and dedication in 2017:
Abbes, B.Linhardt, RobertAbbondanzieri, ElioLionetto, FrancescaAbdollahzadeh Jamalabadi, Mohammad YaghoubLiras, MartaAbel, Steven M.Liu, Xian HuAbidian, MohammadLiu, FeiAbushammala, HatemLiu, YaodongAchilias, Dimitris S.Liu, JinglinAckermann, MaximilianLiu, XianhuAdachi, NaoyaLiu, TingyiAfzal, MohammedLiu, Xu-QingAgrawal, Kumar VaroonLiu, YuanAgubra, Victor AnafoLiu, YanbiaoAguirre, PedroLiu, Chao-LinAhmad, ZeeshanLiu, Shi-XiaAhmed, MaryaLiu, SongAkiba, IsamuLiu, TianboAkitsu, TetsuyaLiu, XifengAlam, ShahriaLizundia, ErlantzAlbo, JonathanLo Re, GiadaAlcoutlabi, MatazLoh, Xian JunAlongi, JennyLohr, TracyAlonso, JavierLolla, DineshAlt, Helmut G.Long, BrianAlvarez-Dominguez, CarmenLongo, SandroAmaro, A.M.Lopez, GartzenAmeduri, BrunoLópez-Lorente, Ángela InmaculadaAmmu, PrhashannaLorenzo Abad, María EncarnaciónAmoako, KagyaLos, PrzemyslawAn, JiaLosada-Perez, PatriciaAndreoni, GiuseppeLou, TiejiongAnnamalai, Pratheep KumarLu, Fa-ChuangAnsari, FarhanLu, YaoAnsón Casaos, AlejandroLu, LuyaoAnthamatten, MitchellLuo, YanhuaAnyszka, RafałLuo, JiangshuiAoki, Ken'ichiLusi, MatteoAoyagi, TakaoLuxenhofer, RobertAparicio Rebollo, Francisco JavierLuzi, FrancescaArabnejad, SaeidLv, AifengAramesh, MortezaLyulin, Alexey V.Aranaz, InmaculadaMa, ZexuArao, YoshihikoMaciejewska, DorotaArgia, KatsuhikoMahoutian, MehrdadArguelles Monal, WaldoMaio, AndreaArosio, PaoloMajor, IanArrate, Emiliano MeaurioMalakooti, Mohammad HArrigo, RossellaMallavia, R.Artesani, AlessiaMalucelli, GiulioAsai, DaisukeMan, DulaAshley, JonManca, Maria LetiziaAthanasios, LadadosManfredi, NorbertoAtrei, AndreaMangayil, RahulAuclair, NicolasMannell, HannaAureliano, ManuelMante, Francis K.Azeloglu, Evren U.Marchesan, SilviaBácskay, IldikóMarcinko, Joseph J.Badea, IldikoMarega, CarlaBadilescu, SimonaMaric, MilanBaghi, HadiMarino, AttilioBai, YingMarpu, Sreekar BabuBaiesi, MarcoMarrocchi, AssuntaBalogh, AttilaMartin-Alfonso, J. E.Bao, ChunhuiMartinez-Salazar, JavierBaranowska-Korczyc, AnnaMasato, DavideBarille, RegisMascia, LenoBarla, ThirupathiMasek, AnnaBarmatov, EvgenyMasoumi, SamiraBarra, GiuseppinaMata, IgnasiBarrau, SophieMatko, VojkoBarriere, FredericMatsukawa, ShingoBashur, ChristopherMatsumoto, AkikazuBattista, EdmondoMatsumoto, MichiakiBaudis, StefanMatsumoto, KozoBayer, Ilker S.Matsumoto, Ken'ichiroBeall, Gary W.Matsumura, ShuichiBear, JosephMatsuoka, HidekiBeard, James D.Matusiak, MalgorzataBedini, EmilianoMauriello, GianluigiBednarz, SzczepanMautner, AndreasBehazin, EhsanMayer, ChristianBella, FedericoMbey, Jean AimeBellini, CostanzoMcGonigal, Paul R.Beltsios, K.McKinley, BrettBenedetti, FabrizioMehrali, MehdiBenito, Juan M.Mekonnen, Tizazu H.Benyahia, BrahimMen, YongjunBéreaux, Y.Meng, F.Bernard, SamuelMenon, AkankshaBerret, Jean-FrançoisMiao, TianxinBertani, RobertaMichels, JulienBeuermann, SabineMichl, Thomas D.Bharadwaj, VivekMiecznikowski, K.Bhattacharya, AmritaMiele, ErmannoBianco, I.D.Milanese, ChiaraBikiaris, DimitriosMiller, Scott F.Bilisik, K.Minakshi, ManickamBinder, WolfgangMiniewicz, AndrzejBiswas, ShaurjoMiranda, KatrinaBlanco, IgnazioMiron, Richard J.Boccia, Antonella CaterinaMirtschin, NikolausBochmann, ManfredMitra, SubhasishBøgwald, JarlMitsuhiro, TerakawaBokias, GeorgiosMitsumata, TetsuBolloli, MarcoMizusaki, MasanobuBolotov, LeonidMłynarczykowska, AnnaBolton, KimMoad, GraemeBoluk, YamanMohammad, ArjmandBon, VolodymyrMolina, MariaBorges, JoãoMolinaro, AntonioBortot, ElianaMonroe, Mary Beth BrowningBose, Ranjita K.Monti, MarcoBose Goswami, SanjuktaMoon, Hong ChulBouropoulos, NikolaosMoon, Doo KyungBowen, JamesMoratti, SteveBoy, RamizMorganti, PierfrancescoBoyd, AnthonyMori, WasukeBraslau, RebeccaMorikawa, AtsushiBrecker, LotharMorimura, ShigeruBrillard, AlainMotta, AntonellaBrooks, Amanda E.Motz, GuenterBrostow, WitoldMozhdehi, DavoudBrüggemann, OliverMugo, SamuelBrunner, AndreasMüller, Alejandro J.Brychcy-Rajska, EwaMüller, WernerBrzeziński, MarekMüller-Buschbaum, PeterBucio Carrillo, EmilioMuñoz, AsunciónBujanovic, BiljanaMuralidhar, AbhiramBuonerba, AntonioMurase, NorioBurkhardt, TheodoreMustafine, RouslanBurnat, BarbaraMutoh, YuichiroBuscaglia, MarcoMystkowska, JoannaCabanelas, Juan CarlosNa, OkpinCabral, JaydeeNagaoka, MasatoCabrales, LuisNagase, YuCaggiano, AntonioNaghashpour, AliCalahorra, YonatanNagy, EndreCallahan, Laura SmithNajorka, JensCametti, C.Nakashima, TakuyaCamposeo, AndreaNakielski, PawelCapacchione, CarmineNanda, HimansuCaputo, Gregory A.Nandwana, VikasCaraglio, MicheleNarayanan, GaneshCarbone, GiuseppeNasiri, MohammadrezaCarette, JérômeNavarro-Fuster, VíctorCarli, StefanoNejad, MojganCarosio, FedericoNejati, SiamakCarrasco, SergioNganga, SaraCarrión-Vilches, Francisco J.Ngo, Truc T.Cartagena-Rivera, AlexanderNguyen, Duc ThanhCaruthers, Marvin H.Nguyen, T. HienCasado-Coterillo, ClaraNicasio, Maria CarmenCasalini, TommasoNicolay, RenaudCaseri, WalterNicoletta, Fiore PasqualeCasettari, LucaNielsen, Line HagnerCassidy, JohnNieminen, KaarloCastell, PereNiidome, TakuroCatanzano, OvidioNishikitani, YoshinoriCausin, ValerioNishiyabu, RyuheiCavicchi, KevinNiskanen, JukkaCech, VladimirNisticò, RobertoCelasco, EdvigeNitschke, MirkoCelik, NumanNoël, MartinCesano, FedericoNoguchi, HiroshiCha, Sung WoonNonappa, NonappaChalioris, ConstantinNonnemacher, Michael R.Chan, Yi-TsuNoonan, KevinChandrasekaran, SwethaNotta-Cuvier, DelphineChang, JiyoungNouranian, SasanChang, You-ImNouvel, CécileChang, Chih-YuNuhn, LutzChang, Chi-jungOaki, YuyaChang, Dong WookObrosov, AlekseiCharas, AnaO'Connell, GraceChase, George G.Oda, TatsuyaChass, Gregory AdamOdelius, KarinChavez, FermanOesterschulze, EgbertChen, Bor-kuanOgawa, NorikoChen, Chuh-YungOgnyanov, ManolChen, Jhy-DerOh, Dongyeop X.Chen, Cheng-LungOhsedo, YutakaChen, JunOk, Jong G.Chen, Min-HueyOkamoto, MasamiChen, Jem-KunOliva, LeoneChen, MingshengOller, EvaChen, JinhuaOllila, SamuliChen, WentaoOmiya, MasakiCheng, XingguoOnwudili, JudeCheng, Chih-ChiaOppelt, Kerstin T.Cheng, PeiOren, YoramCheng, Yang-TseOrte, AngelCheng, Ko-TingOrtenzi, Marco A.Cheng, ZhiqiangOsakada, KohtaroCheong, Yuen-KiÖsterholm, AnnaChiappone, AnnalisaOttonelli, MassimoChiessi, EsterOuyang, LiangqiChladek, GrzegorzOuYang, XiaokunChmielarz, PawełOyoshi, TakanoriCho, Dong-WooPaavilainen, SamiChoi, Kyung-HakPabit, SuzetteChoi, SamjinPalzer, StefanChoi, Hyo-JickPan, GuoqingChoi, Kyu YongPanicker, RajeshChoinopoulos, IoannisPanzarasa, GuidoChou, Shih-FengPapageorgiou, George Z.Chremos, AlexandrosPapavassiliou, Dimitrios V.Chrissopoulou, KiriakiPappalardo, AndreaChruściel, Jerzy J.Pappalardo, DanielaChun, Heung JaeParadiso, RitaChung, Tai-ShungParadiso, ValentinoCicala, GianlucaParaskevopoulou, PatrinaCieplak, MarekParisi, CristianCifre, José G. H.Park, Byung-DaeCinacchi, GiorgioPark, Soo-JinCindemir, UmutPark, Jong-SeokCipelletti, LucaPark, Jang-UngClaramunt, JosepPark, Jong-SeungClochard, Marie-ClaudePark, JeyoungCodispoti, RosamariaParnell, Andrew J.Colby, RalphPassaglia, ElisaComito, Robert J.Paszkiewicz, SandraContreras-Cáceres, RafaelPatel, ShrayeshConway, MichaePatenaude, MathewCooney, RalphPatil, Sunil A.Coppola, BartolomeoPatra, NiranjanCorbatón-Báguena, María-JoséPatrickios, CostasCornell, B.Patrignani, FrancescaCorradi, MarcoPatterson, JenniferCorreia, Ilídio J.Pauer, WernerCosma, PinalysaPaul, WolfgangCosta, RuiPaulsen, HaukeCovello, GiuseppinaPawar, KasturiCovino, RobertoPeleg, OritCranford, StevenPelletier, MathewCui, FangdaPellis, AlessandroCui, JiweiPeng, Chi-HowCui, HaitaoPeng, ZhiliCuoqun, ZhaoPeng, RobertCutrignelli, AnnalisaPeng, XiaoguangD. Shimizu, KenPeng, BoD'accolti, LuciaPerepelyuk, MarynaDagdeviren, CananPérez Amaro, Lucia GabrielaDa'na, EnshirahPerinelli, Diego RomanoDang, Minh TrungPerrin, LaraDarling, SethPersson, IngmarDarmanin, ThierryPeterson, BrandonDas, AmitPeterson, DanielDas, SanjibPeterson, AmyDatta, JanuszPethrick, Richard A.De Grooth, JorisPetkova, DianaDe La Flor Lopez, SilviaPetrović, Zoran S.De la iglesia, ÓscarPhan, HungDe Santis, RobertoPiedade, Ana PaulaDebuigne, AntoinePiletsky, SergeyDeCarlo, Arthur A.Piltonen, PetteriDel Valle, Luis J.Piluso, SusannaDelaney, ColmPilyugina, EkaterinaDelongchamp, DeanPiñol, MilagrosDelplace, VianneyPinteala, MarianaDelpouve, NicolasPinto, ArturDenchev, Zlatan Z.PIRO, BenoitDeterre, RémiPirzada, TahiraDeuss, PeterPispas, StergiosDeutsch, EmericPistone, AlessandroDhanasekar, MPitsikalis, MarinosD'hooge, DagmarPitto-Barry, AnaïsDi, LiPK, DuttaDi Bella, GuidoPlakas, KonstantinosDi Maio, LucianoPlamper, FelixDias, JulianaPlasseraud, LaurentDias, Rolando C.S.Plesa, CalinDickey, MichaelPojman, John A.Dixon, David A.Pokorny, MarekDolgushev, MaximPolavarapu, LakshminarayanaDomenici, ValentinaPolavarapu, LakshminarayanaDominguez, CesarPolimeni, AntonellaDominguez, Juan CarlosPoncet, SébastienDong, WeiPons, JosefinaDong, YixiaoPopielarz, RomanDorozhkin, SergeyPoriel, CyrilDou, JinboPouponneau, PierreDouglas, TimothyPourrahimi, Amir MasoudDow, Wei-PingPrádanos, PedroDowling, DenisPradhan, ShantanuDrozdov, Aleksey D.Prakash, AdithyaDrummond, CarlosPretsch, ThorstenDu, HongjianProcek, MarcinDuan, BinPucci, AndreaDubey, AshishPuglia, DeboraDuffy-Matzner, JettyPuiggali, JordiDumont, Pierre J.J.Puiggalí, J.Duong, Hai MPulati, NuerxidaDutta, BiswanathPunzo, FrancescoDutta, PoulamiPuranik, AmeyDziubla, ThomasPurohit, Prashant K.Ebara, M.Qi, GuangyanEddy, LiyantoQiang, ZheEdirisinghe, MohanQiu, KaiyanEggenhöffner, RobertoQiu, JianhuiEhm, ChristianQuirino, Rafael L.Ehrlich, HermannRabczuk, TimonEjima, HirotakaRabnawaz, MuhammadEllinas, KosmasRades, ThomasElliott, DouglasRaffa, PatrizioElmas, SaitRaftery, RosanneEndruweit, AndreasRagauskas, ArthurErbas, AykutRahme, KamilErdem, EmreRainey, ThomasEscudero, CarlosRajan, RobinEskin, DmitryRamiasa, MelanieEslami, HosseinRana, DipakEspinach, Francesc XavierRao, BalajiEtxeberria, AgustinRao, AshitEvans, ChristopherRao, SmithaFabio Zuluaga, HectorRaposo, MariaFaccio, GretaRastogi, Vibhore KumarFalconer, RobertRaucci, Maria GraziaFally, MartinRavikumar, KumarFan, WeiRebollar, EstherFan, WeizhengRecek, NinaFaraldos, MarisolReece, Michael J.Farris, StefanoRen, FangFarrugia, BrookeRennhofer, HaraldFatyeyeva, KaterynaRestivo, J.Fellows, ChrisRetegi, AloñaFelner, IsraelRicciotti, LauraFeng, HongboRice, Cynthia AnnFeng, QuanyouRiess, GisbertFerhan, Abdul RahimRiggs, Brian C.Fernandes, SuseteRinaldi, AndreaFernandes, Emanuel M.Ritchie, ChristopherFernández, MartaRiziotis, ChristosFerreira Duarte, Ana MarinaRocchigiani, LucaFigueira, RitaRocchitta, GaiaFilippidi, EmmanouelaRocculi, PietroFilpponen, IlariRochat, SébastienFini, StefanoRodriguez-Couto, SusanaFinkenstadt, VictoriaRogers, Simon A.Fiore, VincenzoRojo, JavierFirlak, MelikeRomero, IsabelFischer, DagmarRonca, SaraFischer, HolgerRonca, AlfredoFliedel, ChristopheRondeau-Gagné, SimonFlorczyk, Stephen J.Ronen, AvnerFodor, CsabaRosales, Adrianne M.Follain, NadègeRossi, René M.Fologea, DanielRoth, Stephan V.Fonseca, Ana ClotildeRoth, BernhardFonseca, MariaRousakis, TheodorosFonseca, AnaRoy, SagarFontana, MarcoRuan, HaowenFontenot, Krystal R.Rubenstein, David A.Foraboschi, PaoloRudawska, AnnaFord, JudyRudykh, StephanForget, AurelienRuffo, FrancescoFormela, KrzysztofRuiz Rubio, LeireFossum, EricRupprich, MarcoFox, KateRyabchun, AlexanderFrancesco, NegroRyu, Sung HunFranssen, Maurice C.R.Sabel, TinaFrazier, CharlesSabo, RonaldFreeman, EricSacco, AdrianoFrigione, MariaenricaSacco, PasqualeFrihart, Charles R.Sadrzadeh, MohtadaFroeyen, MatheusSahariah, PriyankaFu, QiangSai, HiroakiFu, LiSaid, Ahmed M.Fu, ZhezhenSaito, ReikoFujisawa, ShujiSakai, ToshioFujita, MorifumiSakurai, KazuoFukuda, TakeshiSalaoru, IuliaFuoco, TizianaSallem-Idrissi, NaimaFurukawa, ReiSalsamendi, MaitaneFurusawa, HiroyukiSalvio, RiccardoGajjar, ChiragSamhaber, W. M.Gallego, SergiSamperi, FilippoGan, Yong X.Sanchiz, JoaquinGanewatta, MitraSandri, GiuseppinaGao, YunxiangSanes, JoséGarcia, IñakiSanjust, EnricoGarcia, AndresSanoh, SeigoGarcía, NuriaSantamaria-Echart, ArantzazuGarcia Ivars, JorgeSantamouris, MattheosGarcia-Gonzalez, Carlos A.Santoni, IlariaGarcía-Martínez, Jesús-MaríaSantulli, CarloGarden, Jennifer ASaralegi, AinaraGaska, KarolinaSarasini, FabrizioGasparini, NicolaSarasua, Jose-RamonGavvalapalli, NagarjunaSashiwa, HitoshiGe, TingSassi, MauroGe, QiSathy, Binulal NelsonGellerstedt, GöranSato, HirotakaGentile, PiergiorgioSato, KimiyasuGeorge, Thomas F.Satriano, CristinaGhassemi, HosseinSavija, BrankoGhiassi, BahmanSawpan, MoyeenuddinGhosh, ArnabScala, AngelaGhoshal, SushantaSchartel, BernhardGhoufi, AzizScherf, UllrichGiacometti, AchilleSchiavo, Sandra LoGigli, MatteoSchirhagl, RomanaGimenez-Pinto, VianneySchlaad, HelmutGindl-Altmutter, WolfgangSchmid, MarkusGiner-Casares, Juan J.Schmid, JochenGiordano, StefanoScholz, CarmenGiovannoli, CristinaSchöneich, MarcGizdavic-Nikolaidis, Marija R.Schubert, StephanieGkountaroulis, DimoklisSchulz, BurkhardtGlüsen, AndreasSchwartzkopf, MatthiasGodeau, GuilhemScoponi, MarcoGoh, Suat HongScribante, AndreaGohs, UweSedo Vegara, JosepGok, OzgulSega, MarcelloGomes, Ana P.Seidel, Rüdiger W.Gómez, Clara M.Sekine, TomohitoGomez-Florit, ManuelSelyanchyn, RomanGomez-Ruiz, SantiagoSemenova, YuliyaGonçalves, M. Sameiro T.Sen, SanghamitraGonzalez, ZoraidaSeo, JihunGonzalez-Elipe, AgustinSerra, AngelsGonzalez-Pedro, VictoriaSeyedin, ShayanGopalan, Sai AnandShafiei, AliGordiichuk, PavloShafiei, MahnazGorrasi, GiulianaShanjani, YaserGoseki, RaitaShanks, Robert A.Goudoulas, ThomasSharma, AnuragGracia, IgnacioSharma, PankajGrassi, GabrieleShavandi, AminGrau, GerdShehata, NaderGreco, AntonioShen, ZhiqiangGu, YibeiShenhar, RoyGu, ChengShestakov, MikhailGubanski, StanislawSheu, Chia-RongGuerriero, GeaShi, XiaoyangGui, MinghuiShi, XueqingGulgunje, PrabhakarShie, Ming-YouGulino, AntoninoShih, Wen-PinGuo, ZhanhuShimada, ShigeruGuo, QiuquanShinde, SudhirkumarGupta, SanjuShiono, TakeshiGupta, MohitShipp, Devon A.Gutekunst, Will R.Shiraishi, YasuhiroGutierrez, JunkalShirvanimoghaddam, KamyarGuvendiren, MuratShishido, AtsushiGuzmán, EduardoShityakov, SergeyGuzmán Solís, EduardoSidorenko, AlexanderHacker, Michael C.Simocko, ChesterHadjiargyrou, MichaelSingh, Pramod K.Hadwiger, LeeSinha, JasmineHager, MartinSinha, AbhijeetHakkarainen, MinnaSiracusa, ValentinaHalada, GarySirvio, JuhoHameed, NisharSisti, LauraHammerl, Jens AndreSixta, HerbertHan, YousooSloma, MarcinHan, MingyuanSloop, JosephHaque, AnwarulSmiatek, JensHara, KenjiSnyder, NicoleHarding, IanSödergård, AndersHardy, John G.Sola, LauraHarmandaris, VagelisSolans-Monfort, XavierHarnois, MaximeSolis-Ramos, EuripidesHarris, Andrew R.Somani, SukrutHassan, MohammadSong, EdwardHassan, El BarbarySong, KenanHassan, Sammer-ulSong, ChangsikHaurie, LaiaSong, ZiyuanHayashi, ShotaroSoria, SilviaHayashi, KenshiSorli, BriceHe, ZhongqiSorrentino, AndreaHe, ZhouyingSotta, PaulHe, HongliangSoulestin, ThibautHe, WenhanSpear, MorwennaHe, JieSperanzini, EmanuelaHeeley, Ellen L.Spina, RobertoHeise, AndreasSrivastava, SamanvayaHeitzmann, MichaelStachewicz, UrszulaHervás-Pérez, J.P.Stassen, IvoHesebeck, OlafStavrinadis, AlexandrosHibino, TakashiStavropoulos, PanagiotisHildner, RichardSteele, Terry W.J.Hiller, MonikaStewart, BeverlyHirai, TomoyasuStolojan, VladHirva, PipsaStone, KariHisada, KenjiStoychev, GeorgiHizal, GürkanStrankowski, MichałHo, Ko-ShanStrano, MichaelHolder, Alvin A.Strube, Oliver I.Holmes, Natalie P.Strzemiecka, BeataHong, TaoStumpe, JoachimHong, LiangStylianakis, Minas M.Hong, Soon HyungSu, An-ChungHönicka, MarkusSu, JingHonus, StanislavSu, ZhouchengHorrocks, A. RichardSubasinghe, Aruna D LHorváth, JuditSugimoto, YoshikiHosseinpourpia, RezaSujecki, SlawomirHou, JingweiSun, HaoHou, ChongSun, LizhiHoujou, HirohikoSun, JingjingHowell, BobSundaram, PaulHsiao, Pai-YiSusmel, SabinaHsiao, Sheng-HueiSuzuki, DaisukeHsu, Hsiu-FuSuzuki, MasahitoHu, ShanghsiuSuzuki, MichioHu, XiaoSwindle-Reilly, Katelyn E.Hu, JimmingSzekely, GyorgyHu, JinlianTabata, YasuhikoHu, YangTabe, ShahramHu, KesongTachibana, YuyaHuang, Chi-HsienTadanaga, KiyoharuHuang, YuchengTakahashi, DaisukeHuang, Chih-FengTakano, ToshiyukiHuang, Chun-JenTakemoto, HiroyasuHuang, QijinTakeuchi, DaisukeHuang, WeiminTamai, ToshiyukiHutchinson, RobinTamminen, TarjaHwang, InchanTamura, AtsushiHwang, YongsungTanaka, KazuoHyun, KyuTanaka, TomonariIandolo, DonataTang, FujianIatrou, HermisTang, LeiIda, DaichiTaniike, ToshiakiIglesias, MartaTarbuk, AnitaIglesias, EmiliaTarres, JoaquimIjiro, KuniharuTeasdale, IanIkehara, TakayukiTenorio-Alfonso, AdriánIniesta Valcarcel, JesusTeramoto, YoshikuniInomata, KatsuhiroTeramoto, NaozumiInvernizzi, MartaTerashima, TakayaIohara, DaisukeTercjak, AgnieszkaIordanskii, AlexeyTextor, TorstenIrie, MasaoThakur, Vijay KumarIshige, RyoheiThérien-Aubin, HéloïseIshikawa, RyutaThielmann, JulianIshikawa, DataroThomas, SamuelItagaki, HideyukiThomas, T. JohnItkis, Mikhail E.Tijing, LeonardIto, ShingoTirloni, EricaIto, TaichiToal, VincentIwan, AgnieszkaTodoroki, AkiraJacak, JarosławTohmura, Shin-ichiroJain, GauravTomaiuolo, GiovannaJajam, Kailash CTondi, GianlucaJamali, SafaTonelli, Alan EdwardJan, Jeng-shiungTorres, CristianaJanas, DawidTorroba, TomasJanczarek, MonicaTortora, LucaJang, Kyung-InTosello, GuidoJarvis, KarynTotsingan, FilbertJavadi, AliTrabulo, SaraJayamohan, HarikrishnanTrakakis, GeorgeJayant, Rahul DevTremblay, Marie-LaurenceJayaram, Dhanya T.Trif, MonicaJayasinghe, Suwan Trifol, JonJeeyoung, YooTrotta, FrancescoJeon, Ju-WonTruong Phuoc, NghiaJeong, SunghoonTruss, RowanJeong, Mi-YunTrusso Sfrazzetto, GiuseppeJérôme, ValérieTsakalof, AndreasJesionowski, TeofilTsarkova, Larisa A.Jha, Kshitij C.Tsitsilianis, ConstantinosJia, WeiweiTsujimoto, TakashiJiang, LiuweiTubiana, LucaJiang, YiTunesi, MartaJiang, ShaohuaTung, Chih-KuanJiang, XianTura, AndreaJofre-Reche, Jose AntonioTzounis, LazarosJohn, LukaszUbrani Mruthunjayappa, Manu PatelJones, DavidUemura, KazuhiroJoo, Hong-GuUneyama, TakashiJoshi, NiravkumarUppstu, PeterJoshi, VijayaUrbanchek, Melanie G.Jung, Hyo SungUrbina-Blanco, César A.Jung, Ho-SupUsher, Tedi-MarieJung, JaehanUyama, HiroshiJung, Kyung-HyeUzunlar, ErdalJung, Young DoVagin, Mikhail Yu. Kadakowa, Jun-ichiVan Herwijnen, Hendrikus W.G.Kadar, RolandVan Herwijnen, HendrikKafarski, PawełVan Nhien, Albert NguyenKai, DanVan Steenberge, Paul Hm M.Kakkar, AshokVancaeyzeele, CédricKalali, Ehsan NaderiVankayala, RavirajKalantar-Zadeh, KouroshVasile, CorneliaKallitsis, Joannis K.Vautard, FredericKambhampati, Siva PramodhVelotta, RaffaeleKan, Chi-waiVenditti, IoleKanakubo, ToshiyukiVenkataraman, ShrinivasKang, Sang WookVerduzco, RafaelKang, InpilVerma, SannyKang, LifengVermaas, Joshua V.Karabiyik, UfukVernardou, DimitraKarger-Kocsis, JózsefVijayavenkataraman, SanjairajKaron, KrzysztofVila, Xosé A.Kasem, Kasem K.Villain, SylvieKashi, SimaVirnau, PeterKasoju, NareshVisco, AnnamariaKathuria, HimanshuVlachopoulos, JohnKatogi, HideakiVoliani, ValerioKaunas, Roland R.Vouyiouka, Stamatina N.Kawaguchi, HarumaWaheed, SalwanKawamura, AkifumiWalach, WojciechKawamura, IzuruWalder, LorenzKeip, Marc-AndreWaldmeier, FelixKeller, BrandisWalker, JulianKerch, GarryWalther, FrankKeul, HelmutWang, Jian-WenKhaldi, AlexandreWang, Yun-MingKhan, AnzarWang, WenxinKhandaker, MorshedWang, LiliKhare, KetanWang, XiaofengKhazi, Mohammed IqbalWang, QinyiKidoaki, SatoruWang, Wen-YiKiefer, RudolfWang, Peng-YuanKihara, NobuhiroWang, MingchaoKim, KukjooWang, Tsu-WeiKim, DaewonWang, LiangKim, JongseongWang, Dong HwanKim, Jae-HongWang, HaoyuKim, SooyoungWang, De-YiKim, HoonWang, XinKim, Byung KyuWang, LizhuKim, Il TaeWang, XiaoguangKim, Hak-YongWebber, GrantKim, Gue-hyunWei, JiaKim, Pyung-HwanWei, QiangKim, SeokminWei, HuaKiryukhin, Maxim V.Weng, LihuiKlaehn, John R.Więckiewicz, MieszkoKlein, RolandWiesbrock, FrankKlonos, PanagiotisWilhelm, StefanKlotz, AlexWillerth, Stephanie MKnetsch, Menno L.W.Williams, Kathryn R.Koetz, JoachimWillis, AshleyKohri, MichinariWinlove, PeterKohsaka, YasuhiroWinnacker, MalteKõiva, RistoWischke, ChristianKoizumi, ToshioWitkowski, ArturKolbe, JanaWojkiewicz, Jean-LucKoller, MartinWolak, MasonKonkolewicz, DominikWoo, Han YoungKonnerth, JohannesWoo, EamorKootala, SujitWood, DelilahKoren, KlausWoodward, Robert T.Körstgens, VolkerWu, JianboKortschot, Mark T.Wu, TaoKostakis, GeorgeWu, Ren-JangKotobuki, MasashiWu, Tzi-YiKotsikorou, EvangeliaWu, Kevin C.-W.Koumba-Yoya, GeorgesWu, Li-ChenKowalczuk, MarekWu, Yu-TseKoźlecki, TomaszWyman, IanKozliak, Evguenii I.Wysokowski, MarcinKressler, JörgXavier, JoséKrishnan, LathaXi, WeixianKropp, MartinaXiang, ChunhuiKruk, MichalXIANG, YuanKrystofiak, TomaszXie, XinKuang, HuihuiXu, ZelinKubo, TakuyaXu, Jun-TingKubo, MasatakaXu, JianweiKuckling, DirkXu, YanyiKucuk, IsrafilXu, XiaochuanKujawski, WojciechXu, JiangtaoKulzer, FlorianXu, XiaoxueKumar, SubhalakshmiYabu, HiroshiKumar, SudheeshYamada, ToruKunjukunju, SangeethaYan, LiangKuo, Shiao-WeiYan, LiboKuo, Chung-WenYan, DayunKuroda, KosukeYan, NingKuroda, YoshiyukiYanagida, MasatoshiKusano, ShuheiYang, ChengKustov, LeonidYang, Hung-WeiKuwabara, JunpeiYang, GuangKwon, Young-WanYannakopoulou, KonstantinaKwon, Tae-yubYao, QingqingLa Mantia, Francesco PaoloYao, Zi-JianLabidi, JalelYao, ShanshanLade, HarshadYasuda, TakeshiLadero, MiguelYates, Matthew Z.Lai, Jui-yangYe, LongLakard, BorisYe, JunLamprou, DimitriosYe, QunLanceros-Méndez, SenentxuYe, ZhouLang, GregorYeager, JohnLange, HeikoYee, Shannon K.Lantada, Andres DiazYeh, Yin-TingLaPointe, AnneYeh, Jui-MingLara-Sanchez, AgustinYeo, HyeonukLaroche, Céline LarocheYeong, Wai YeeLarraneta Landa, EnekoYin, JunLarrea, Edurne S.Ying, HanzeLauricella, MarcoYoon, HyeonseokLavina, SandraYoshikawa, KenichiLavorgna, MarinoYou, JungmokLazzara, GiuseppeYouk, Ji HoLazzarato, LorettaYousefi, Azizeh-MitraLeblanc, Roger M.Yousif, BelalLebon, FrédéricYu, Yuan-HsiangLecomte, PhilippeYu, JianLee, Jin-KyunYu, LeonLee, Shyi-LongYu, Deng-GuangLee, Sang-JoonYu, XinjunLee, Bor-ShiunnYu, JiayiLee, Wen-JauYu, JaecheulLee, Jae-SukYu, ZiniuLee, SangminYu, YingjieLee, SunghwanYu, WeilingLee, Yun-HanYuan, ZhihongLee, Jung-JaeYuan, YonganLee, Bun YeoulYuan, JunqiLee, JaewoongZaccone, AlessioLee, Li-TingZaręba, JanLee, Wen-YaZarejousheghani, MashaalahLéger, BastienZavarise, GiorgioLemma, Solomon MengistuZechel, StefanLemma, SolomonZekonyte, JurgitaLeporatti, StefanoZeng, HuaqiangLepore, MariaZentel, RudolfLesiak, PiotrZeugolis, DimitiosLewis, RandolphZha, ShangwenLi, MeichunZhang, QichunLi, KaichangZhang, XuefengLi, Robert Kwok-YiuZhang, WujieLi, YingZhang, YuLi, PingZhang, YongLi, Jiao JiaoZhang, HongxiLi, JiantongZhang, MengLi, LongyuZhang, YumiaoLi, HangZhang, QianqianLi, Min-HuiZhang, SanliangLi, ZibiaoZhang, YingyueLi, QianZhao, BoxinLi, AnlongZhao, XinxinLi, ShuaiZhao, WeifengLi, YiwenZhao, HongLi, JinghaoZheng, QingbinLi, YifeiZheng, WeiweiLiakos, Ioannis L.Zhi, FangLiang, KangZhong, TuhuaLiao, Shu-ChuanZhou, DezhongLiaw, Chya-YanZhu, XiangweiLignola, Gian PieroZhu, RongLim, KhoonZhu, MeifangLima, CarmineZhu, WeiLin, Yang-weiZhu, JiadengLin, Hung-yinZi, YunlongLin, XiaojieZinchenko, AnatolyLin, HaijingZornoza, BeatrizLin, HaiqingZou, QiongjingLin, Ching HsuanZuccaccia, CristianoLin, Tsung-HsienZuo, JianLing, Shengjie


